# Risk factors and development of a nomogram prediction model for urinary dysfunction after laparoscopic radical surgery for rectal cancer

**DOI:** 10.3389/fsurg.2026.1911971

**Published:** 2026-08-11

**Authors:** Baoming Zhang, Wenchao Shi, Shanting Gao

**Affiliations:** 1 Department of Gastrointestinal Surgery, The First People’s Hospital of Lianyungang, Lianyungang, Jiangsu, China; 2 Department of General Surgery, Horgos People's Hospital, Horgos, Ili Kazakh Autonomous Prefecture, Xinjiang Uygur Autonomous Region, China

**Keywords:** laparoscopic radical surgery, nomogram, prediction model, rectal cancer, risk factors, urinary dysfunction

## Abstract

**Background:**

Urinary dysfunction is a relatively common functional complication after laparoscopic radical surgery for rectal cancer and may affect postoperative recovery and quality of life. This study aimed to identify risk factors for urinary dysfunction after laparoscopic radical rectal cancer surgery and to develop a nomogram prediction model to facilitate early identification of high-risk patients.

**Methods:**

A total of 392 patients who underwent laparoscopic radical surgery for rectal cancer between January 2021 and December 2025 were retrospectively included. Patients were grouped according to the occurrence of postoperative urinary dysfunction and randomly divided into a training cohort and a validation cohort at a ratio of 7:3. Demographic, tumor-related, and perioperative variables were collected. Univariable and multivariable logistic regression analyses were performed to identify independent predictors, and a nomogram model was constructed. Model performance was evaluated using receiver operating characteristic curves, calibration curves, the Hosmer-Lemeshow test, and decision curve analysis.

**Results:**

Among the 392 patients, 84 developed postoperative urinary dysfunction, with an incidence of 21.4%. Multivariable logistic regression analysis showed that age ≥65 years, male sex, **t**umor distance from the anal verge ≤5 cm, intraoperative fluid rate ≥10 mL/kg/h, neoadjuvant chemoradiotherapy, documented incomplete pelvic autonomic nerve preservation, and postoperative urinary tract infection were independent factors associated with postoperative urinary dysfunction. A nomogram was constructed based on these predictors. The area under the curve was 0.864 (95% CI: 0.803–0.926) in the training cohort and 0.856 (95% CI: 0.774–0.938) in the validation cohort. Calibration curves and the Hosmer-Lemeshow test indicated good model fit, and decision curve analysis showed favorable clinical net benefit.

**Conclusion:**

Age, sex, tumor distance from the anal verge, intraoperative fluid rate, neoadjuvant chemoradiotherapy, pelvic autonomic nerve preservation, and postoperative urinary tract infection were closely associated with urinary dysfunction after laparoscopic radical surgery for rectal cancer. The nomogram based on these factors showed good predictive performance and may help identify high-risk patients, guide urinary catheter management, and support postoperative follow-up.

## 1 Introduction

Rectal cancer is one of the most common malignancies of the digestive system (1). With the development of the concept of total mesorectal excision (TME), laparoscopic techniques, and neoadjuvant treatment strategies, local control and long-term survival in patients with rectal cancer have continuously improved (2, 3). Against the background of improved oncological outcomes, postoperative functional preservation and quality of life have gradually become important aspects of comprehensive rectal cancer management (4, 5). In addition to low anterior resection syndrome and sexual dysfunction, urinary dysfunction is also a relatively common but easily underestimated functional complication after radical surgery for rectal cancer (6, 7). Patients may present with dysuria, urinary retention, increased postvoid residual urine volume, failure of catheter removal, repeated catheterization, or the need for persistent catheterization after discharge. These conditions not only impair postoperative recovery experience but may also increase the risk of urinary tract infection, prolong hospital stay, and reduce postoperative quality of life (8–10).

The mechanisms underlying urinary dysfunction after rectal cancer surgery are complex, among which pelvic autonomic nerve injury is considered an important cause (11). The operative field of radical rectal cancer surgery is located deep in the pelvis and is adjacent to important autonomic nerve structures, including the hypogastric nerves, pelvic splanchnic nerves, and pelvic plexus. In patients with low rectal cancer or complex local anatomy, surgical manipulation may increase the risk of pelvic autonomic nerve injury, thereby affecting bladder sensation and detrusor contractile function (12, 13). Previous studies have suggested that some patients may develop varying degrees of urinary dysfunction after rectal cancer treatment, and intraoperative identification and preservation of the pelvic autonomic nerves may help reduce the risk of postoperative bladder dysfunction (14, 15). Therefore, early perioperative identification of patients at high risk for postoperative urinary dysfunction, followed by individualized catheter management, bladder function training, and follow-up intervention, is an important issue in functional recovery management after rectal cancer surgery.

In recent years, several studies have attempted to develop scoring systems to assess the risk of bladder dysfunction after laparoscopic rectal cancer surgery, and others have investigated risk factors for postoperative urinary retention after colorectal surgery (16–18). However, existing studies have mainly focused on single-factor analyses or conventional scoring methods, and individualized prediction models for urinary dysfunction after laparoscopic radical surgery for rectal cancer remain relatively limited. A nomogram can integrate multiple clinical factors and visually estimate an individual patient's risk of a specific outcome, providing good clinical interpretability and practical value. Therefore, this study retrospectively analyzed clinical data from patients who underwent laparoscopic radical surgery for rectal cancer, screened for factors associated with postoperative urinary dysfunction, and developed a nomogram prediction model, aiming to provide a reference for early identification of high-risk patients, optimization of catheter removal strategies, and promotion of postoperative functional recovery.

## 2 Materials and methods

### 2.1 Study design and ethics

This was a single-center retrospective cohort study. Patients who underwent laparoscopic radical surgery for rectal cancer in the Department of Gastrointestinal Surgery between January 2021 and December 2025 were included. This study was conducted in accordance with the principles of the Declaration of Helsinki (19) and was approved by the hospital ethics committee. Owing to the retrospective nature of the study and the exclusive use of previously collected clinical data without any intervention in patient treatment, all personal information was anonymized during data analysis.

### 2.2 Study population

The inclusion criteria were as follows: (1) pathologically confirmed primary rectal adenocarcinoma after surgery; (2) age ≥18 years; (3) receipt of laparoscopic radical surgery for rectal cancer, including low anterior resection, ultralow anterior resection, or abdominoperineal resection; and (4) complete perioperative clinical data and postoperative urinary function assessment data. The exclusion criteria were as follows: (1) pre-existing definite urinary dysfunction before surgery, including preoperative urinary retention, long-term indwelling catheterization, intermittent catheterization, neurogenic bladder, urethral stricture, severe benign prostatic hyperplasia, or lower urinary tract symptoms requiring long-term medication or surgical intervention; (2) concomitant bladder tumor, prostate cancer, or other urological diseases that could significantly affect urinary function; (3) concomitant malignancies of other pelvic organs or simultaneous complex combined organ resection; (4) severe missing perioperative data; (5) postoperative death or inability to assess urinary function; and (6) unavailable or insufficient operative documentation for determining pelvic autonomic nerve-preservation status.

### 2.3 Definition of postoperative urinary dysfunction

In this study, postoperative urinary dysfunction was defined as the occurrence of any of the following conditions after catheter removal following laparoscopic radical surgery for rectal cancer (17, 20): (1) inability to void spontaneously or dysuria requiring repeated catheterization or reinsertion of an indwelling catheter; (2) a postvoid residual urine volume ≥200 mL on an initial bladder ultrasound measurement after spontaneous voiding and on a second measurement after the next spontaneous void; (3) the need for persistent indwelling catheterization or intermittent catheterization at discharge; or (4) documented dysuria, urinary retention, or incomplete bladder emptying requiring medication or bladder function training. When immediate catheterization was performed because of inability to void or symptomatic urinary retention, a second postvoid residual urine measurement was not performed, and the patient was classified according to criterion (1). The cutoff value of 200 mL was adopted from a previous study of patients undergoing laparoscopic rectal cancer surgery (17) and was consistent with the postoperative bladder-management protocol of our institution. Urological consultation was requested in cases of persistent inability to void, postvoid residual urine volume ≥200 mL requiring catheterization, repeated catheterization, or persistent dysuria after initial management. Urological consultation itself was not used as a criterion for classifying postoperative urinary dysfunction. Patients were divided into the urinary dysfunction group and the non-urinary dysfunction group according to the occurrence of postoperative urinary dysfunction.

### 2.4 Observed variables and variable assignment

Based on clinical availability and the purpose of this study, 13 candidate predictors were included: age, sex, diabetes mellitus, tumor distance from the anal verge, neoadjuvant chemoradiotherapy, pelvic autonomic nerve preservation, operation time, intraoperative blood loss, intraoperative fluid rate, catheterization duration, postoperative urinary tract infection, preoperative albumin, and neutrophil-to-lymphocyte ratio (NLR). Continuous variables were retained in their original continuous form for descriptive analysis. For logistic regression analysis and nomogram development, continuous predictors were categorized as follows: age, <65 versus ≥65 years; tumor distance from the anal verge, ≤5 versus >5 cm; operation time, <180 versus ≥180 min; intraoperative blood loss, <100 versus ≥100 mL; intraoperative fluid rate, <10 versus ≥10 mL/kg/h; catheterization duration, ≤5 versus >5 days; preoperative albumin, ≥35 versus <35 g/L; and NLR, <3 versus ≥3. Preoperative albumin and NLR were defined as the most recent blood test results obtained after admission and before surgery. Intraoperative fluid rate was calculated based on the total intraoperative fluid volume recorded in the anesthesia record, patient body weight, and operation time, using the following formula: intraoperative fluid rate (mL/kg/h) = total intraoperative fluid volume (mL)/body weight (kg)/operation time (h). Pelvic autonomic nerve preservation was determined according to the operative records. Cases with explicit documentation of preservation of the hypogastric nerves, pelvic plexus, or pelvic autonomic nerves were classified as “documented preservation,” whereas cases with explicit documentation of incomplete preservation were classified as “documented incomplete preservation.”

### 2.5 Data splitting and model construction

Patients were randomly divided into a training cohort and a validation cohort at a ratio of 7:3. The training cohort was used to screen predictors and construct the nomogram model, whereas the validation cohort was used to evaluate model performance. In the training cohort, univariable logistic regression analyses were first performed to examine the association between each candidate predictor and postoperative urinary dysfunction. Variables with *P* < 0.05 in the univariable analyses were included in the multivariable logistic regression analysis. Predictors that remained statistically significant at *P* < 0.05 in the multivariable analysis were included in the final nomogram model. The nomogram was constructed using R software.

### 2.6 Model evaluation and validation

Model discrimination was evaluated using the receiver operating characteristic (ROC) curve and the area under the curve (AUC). Model calibration was assessed using calibration curves and the Hosmer-Lemeshow goodness-of-fit test, with *P* > 0.05 indicating no evidence of poor model fit. The clinical utility of the model was evaluated using decision curve analysis (DCA). The training cohort was used for model construction, whereas the validation cohort was used for internal validation. Model discrimination, calibration, and clinical net benefit were evaluated in both cohorts.

### 2.7 Statistical analysis

Statistical analyses were performed using SPSS version 26.0 and R version 4.5.0. Continuous variables were retained in their original continuous form for descriptive analysis, expressed as mean ± standard deviation, and compared between groups using the independent-samples t-test. For logistic regression analysis and nomogram development, continuous predictors were categorized according to the cutoff values specified in Section 2.4. Categorical variables were expressed as numbers and percentages and compared using the *χ*^2^ test or Fisher's exact test, as appropriate. Multicollinearity was assessed using the variance inflation factor (VIF), with VIF >5 indicating potential multicollinearity. All tests were two-sided, and *P* < 0.05 was considered statistically significant.

## 3 Results

### 3.1 Incidence of postoperative urinary dysfunction and comparison of general characteristics

A total of 392 patients who underwent laparoscopic radical surgery for rectal cancer were included in this study. Among them, 84 patients developed postoperative urinary dysfunction, corresponding to an incidence of 21.4%, whereas 308 patients did not develop postoperative urinary dysfunction, accounting for 78.6%. Compared with the non-urinary dysfunction group, the urinary dysfunction group had higher proportions of male patients, patients with diabetes mellitus, patients who received neoadjuvant chemoradiotherapy, patients with documented incomplete pelvic autonomic nerve preservation, and patients with postoperative urinary tract infection. The distribution of surgical procedures also differed significantly between the two groups, and tumor distance from the anal verge was shorter in the urinary dysfunction group (all *P* < 0.05). No statistically significant differences were observed in age, operation time, intraoperative blood loss, catheterization duration, NLR, intraoperative fluid rate, or preoperative serum albumin between the two groups (all *P* > 0.05) (Table 1).

**Table 1:** Comparison of general and perioperative characteristics between patients with and without postoperative urinary dysfunction.

Variables	Total (*n* = 392)	No urinary dysfunction (*n* = 308)	Urinary dysfunction (*n* = 84)	P value
Age, years	65.6 ± 9.3	65.3 ± 9.7	66.6 ± 7.6	0.265
Sex, n (%)				0.007
Female	177 (45.2)	150 (48.7)	27 (32.1)	
Male	215 (54.8)	158 (51.3)	57 (67.9)	
Diabetes mellitus, n (%)				0.02
No	342 (87.2)	275 (89.3)	67 (79.8)	
Yes	50 (12.8)	33 (10.7)	17 (20.2)	
Surgical procedure, n (%)				<0.001
Low anterior resection	195 (49.7)	181 (58.8)	14 (16.7)	
Ultralow anterior resection	111 (28.3)	82 (26.6)	29 (34.5)	
Abdominoperineal resection	86 (21.9)	45 (14.6)	41 (48.8)	
Distance of tumor from the anal verge, cm	5.4 ± 2.2	5.9 ± 2.2	3.7 ± 1.4	<0.001
Operation time, min	202.5 ± 41.2	200.4 ± 42.2	210.1 ± 36.9	0.054
Intraoperative blood loss, mL	74.9 ± 38.1	73.3 ± 39.3	80.8 ± 32.8	0.107
Catheterization duration, days	4.5 ± 1.1	4.5 ± 1.0	4.4 ± 1.6	0.483
Neutrophil-to-lymphocyte ratio	2.9 ± 1.0	2.9 ± 1.0	3.0 ± 0.8	0.628
Intraoperative fluid rate, mL/kg/h	7.4 ± 1.7	7.4 ± 1.5	7.5 ± 2.1	0.663
Preoperative serum albumin, g/L	41.0 ± 5.4	41.0 ± 5.1	40.8 ± 6.1	0.77
Neoadjuvant chemoradiotherapy, n (%)				<0.001
No	274 (69.9)	238 (77.3)	36 (42.9)	
Yes	118 (30.1)	70 (22.7)	48 (57.1)	
Pelvic autonomic nerve preservation, n (%)				<0.001
Preservation	361 (92.1)	297 (96.4)	64 (76.2)	
Incomplete preservation	31 (7.9)	11 (3.6)	20 (23.8)	
Postoperative urinary tract infection, n (%)				<0.001
No	375 (95.7)	305 (99.0)	70 (83.3)	
Yes	17 (4.3)	3 (1.0)	14 (16.7)	

Continuous variables are presented as mean ± standard deviation, and categorical variables as number (percentage).

### 3.2 Univariable logistic regression analysis

Univariable logistic regression analysis was performed in the training cohort. The results showed that age ≥65 years, male sex, diabetes mellitus, tumor distance from the anal verge >5 cm, operation time ≥180 min, intraoperative blood loss ≥100 mL, catheterization duration >5 days, NLR ≥3, intraoperative fluid rate ≥10 mL/kg/h, preoperative albumin <35 g/L, neoadjuvant chemoradiotherapy, documented incomplete pelvic autonomic nerve preservation, and postoperative urinary tract infection were associated with postoperative urinary dysfunction (all *P* < 0.05) (Table 2).

**Table 2:** Univariate logistic regression analysis of postoperative urinary dysfunction in the training cohort.

Variables	OR (95%CI)	P value
Age		
<65 years	Reference	
≥65 years	5.113 (2.509,10.423)	<0.001
Sex		
Female	Reference	
Male	2.037 (1.094,3.794)	0.025
Diabetes mellitus		
No	Reference	
Yes	2.948 (1.368,6.351)	0.006
Tumor distance from the anal verge		
≤5 cm	Reference	
＞5 cm	0.161 (0.077,0.337)	<0.001
Operation time		
<180 mintues	Reference	
≥180 mintues	2.155 (1.075,4.320)	0.03
Intraoperative blood loss		
<100 mL	Reference	
≥100 mL	1.915 (1.052,3.485)	0.033
Catheterization duration		
≤5 days	Reference	
>5 days	5.981 (2.119,16.886)	0.001
NLR		
<3	Reference	
≥3	2.734 (1.461,5.116)	0.002
Intraoperative fluid rate		
<10 mL/kg/h	Reference	
≥10 mL/kg/h	5.634 (2.575,12.330)	<0.001
Preoperative albumin		
≥35 g/L	Reference	
<35 g/L	2.354 (1.140,4.858)	0.021
Neoadjuvant chemoradiotherapy		
No	Reference	
Yes	5.347 (2.852,10.024)	<0.001
Pelvic autonomic nerve preservation		
Documented preservation	Reference	
Incomplete preservation	9.463 (3.564,25.127)	<0.001
Postoperative urinary tract infection		
No	Reference	
Yes	20.279 (5.489,74.915)	<0.001

OR, odds ratio; CI, confidence interval; NLR, neutrophil-to-lymphocyte ratio.

### 3.3 Multicollinearity assessment

The multicollinearity assessment showed that the VIF values of all candidate predictors ranged from 1.03 to 1.30, with a mean VIF of 1.16. All values were below 5, indicating no evidence of substantial multicollinearity among the candidate predictors. Therefore, these variables were considered suitable for inclusion in the multivariable logistic regression analysis (Supplementary Table S1).

### 3.4 Multivariable logistic regression analysis

Multivariable logistic regression analysis showed that age ≥65 years, male sex, tumor distance from the anal verge ≤5 cm, intraoperative fluid rate ≥10 mL/kg/h, neoadjuvant chemoradiotherapy, documented incomplete pelvic autonomic nerve preservation, and postoperative urinary tract infection were independent factors associated with urinary dysfunction after laparoscopic radical surgery for rectal cancer (Table 3).

**Table 3:** Multivariable logistic regression analysis of postoperative urinary dysfunction in the training cohort.

Variable	*β*	SE	Wald *χ*^2^	OR (95% CI)	P value
Age					
<65 years				Reference	
≥65 years	1.506	0.481	9.797	4.510 (1.756,11.585)	0.002
Sex					
Female				Reference	
Male	0.934	0.438	4.550	2.545 (1.079,6.007)	0.033
Diabetes mellitus					
No				Reference	
Yes	0.809	0.583	1.929	2.247 (0.717,7.041)	0.165
Tumor distance from the anal verge					
≤5 cm				Reference	
＞5 cm	−0.910	0.461	3.897	0.403 (0.163,0.993)	0.048
Operation time					
<180 minutes				Reference	
≥180 minutes	0.010	0.525	0.001	1.010 (0.361,2.823)	0.985
Intraoperative blood loss					
<100 mL				Reference	
≥100 mL	0.682	0.448	2.323	1.979 (0.823,4.758)	0.127
Catheterization duration					
≤5 days				Reference	
>5 days	0.830	0.797	1.086	2.294 (0.481,10.942)	0.298
NLR					
<3				Reference	
≥3	0.863	0.456	3.580	2.370 (0.969,5.795)	0.059
Intraoperative fluid rate					
<10 mL/kg/h				Reference	
≥10 mL/kg/h	1.733	0.599	8.369	5.657 (1.748,18.302)	0.004
Preoperative albumin					
≥35 g/L				Reference	
<35 g/L	0.780	0.528	2.182	2.182 (0.775,6.145)	0.140
Neoadjuvant chemoradiotherapy					
No				Reference	
Yes	0.961	0.464	4.285	2.614 (1.052,6.492)	0.038
Pelvic autonomic nerve preservation					
Documented preservation				Reference	
Incomplete preservation	1.922	0.687	7.829	6.833 (1.778,26.259)	0.005
Postoperative urinary tract infection					
No				Reference	
Yes	3.526	1.109	10.106	33.971 (3.866,298.531)	0.001

OR, odds ratio; CI, confidence interval; SE, standard error.

### 3.5 Establishment of the nomogram prediction model

**Figure 1:**
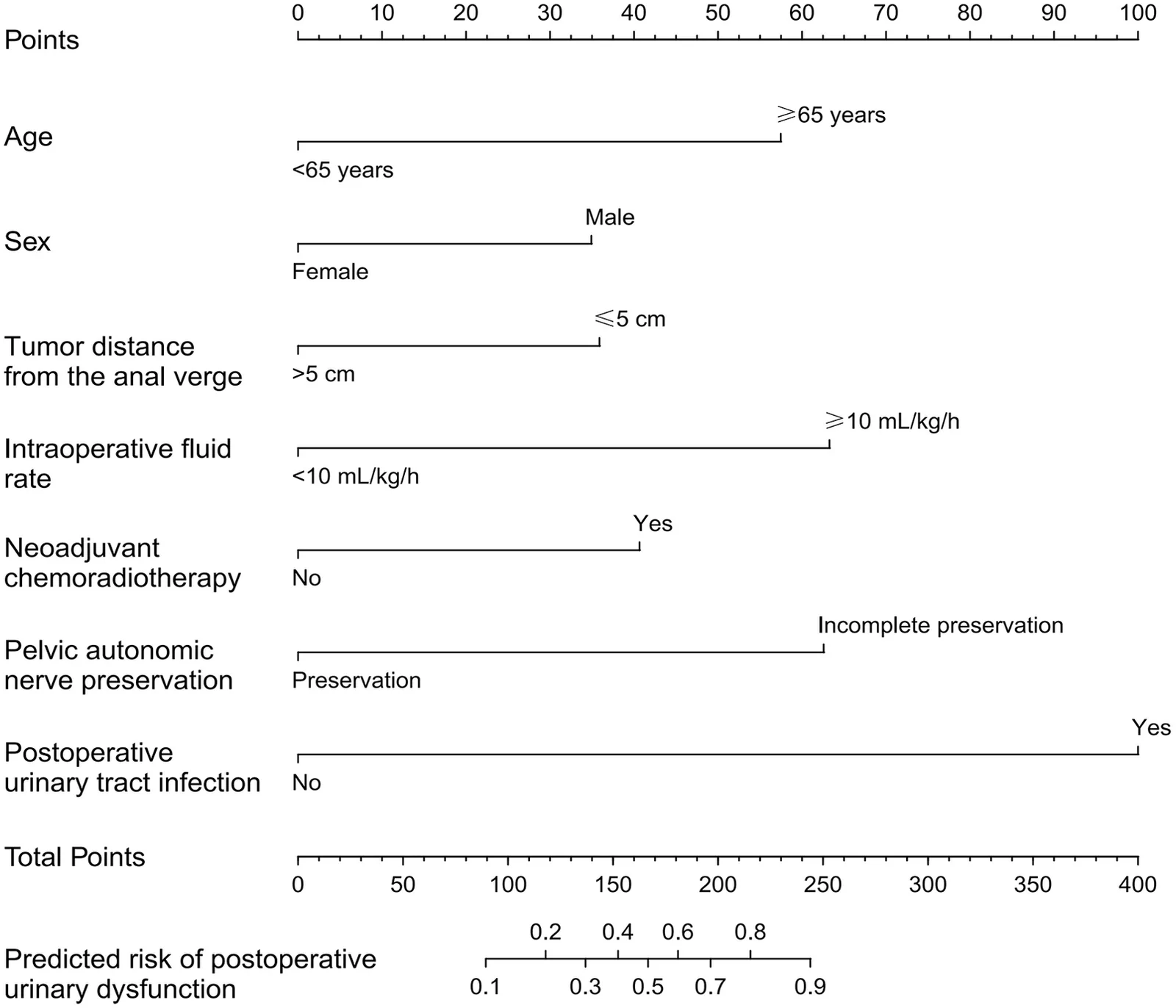
Nomogram for predicting postoperative urinary dysfunction after laparoscopic radical rectal cancer surgery. The nomogram was constructed using independent predictors identified by multivariable logistic regression, including age, sex, tumor distance from the anal verge, intraoperative fluid rate, neoadjuvant chemoradiotherapy, pelvic autonomic nerve preservation, and postoperative urinary tract infection. The points assigned to each predictor are summed to obtain a total score, which is mapped to the probability scale to estimate the individual probability of postoperative urinary dysfunction.

Based on the results of multivariable logistic regression analysis in the training cohort, seven independent predictors, including age, sex, tumor distance from the anal verge, intraoperative fluid rate, neoadjuvant chemoradiotherapy, pelvic autonomic nerve preservation, and postoperative urinary tract infection, were included to construct a nomogram model for predicting the risk of urinary dysfunction after laparoscopic radical surgery for rectal cancer. In the nomogram, each predictor corresponds to a specific score. The scores of all variables are summed to obtain a total score, and the corresponding risk scale is then used to estimate the individualized risk of postoperative urinary dysfunction (Figure 1). The detailed point assignment of each predictor is shown in Supplementary Table S2. For clinical application, the score corresponding to each predictor is determined from the “Points” axis, and the seven scores are summed to obtain the total score. The total score is then mapped to the probability scale to estimate the individual probability of postoperative urinary dysfunction, with a higher total score indicating a higher predicted probability.

### 3.6 Discrimination of the nomogram prediction model

**Figure 2:**
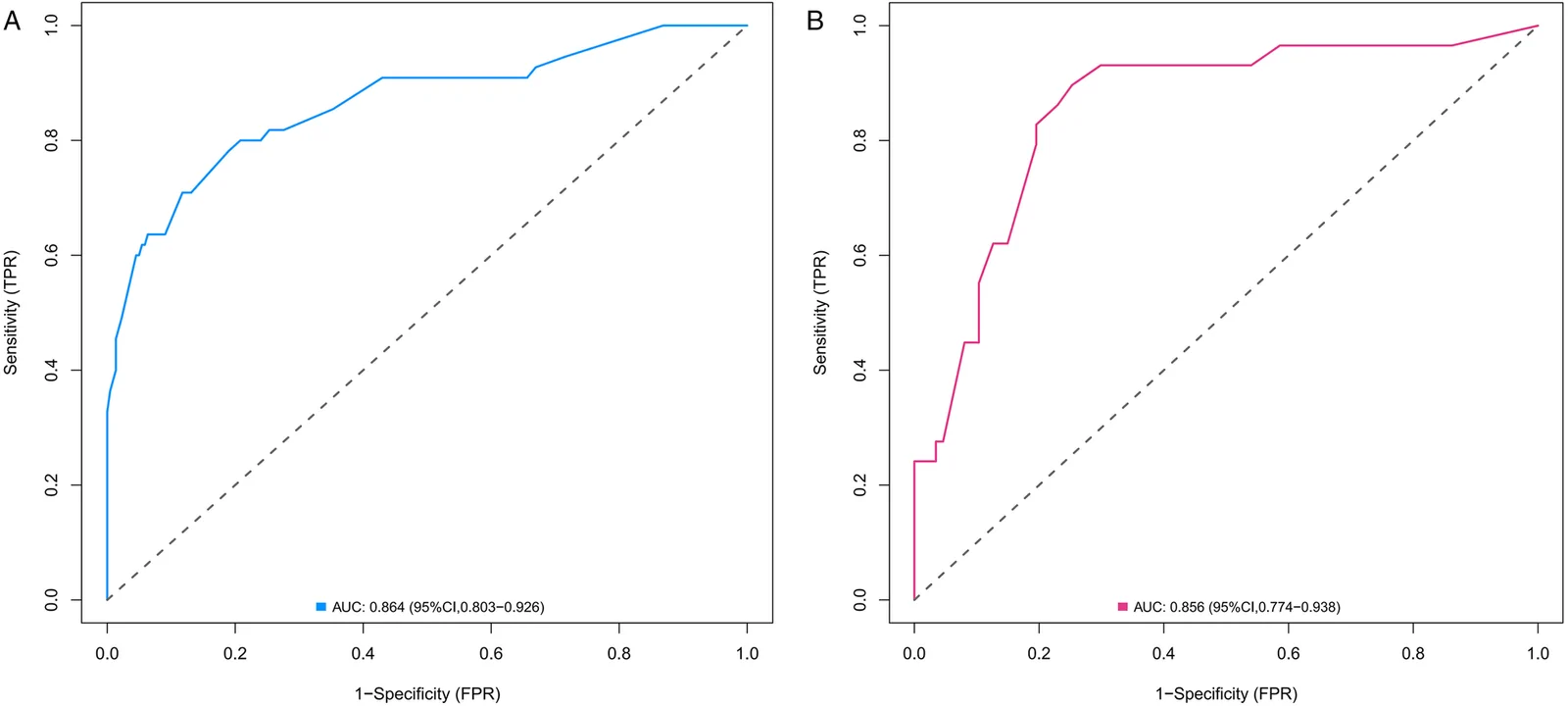
Roc curves of the nomogram for predicting postoperative urinary dysfunction in the training cohort **(A)** and validation cohort **(B).**

ROC curve analysis showed that the AUC of the nomogram model was 0.864 (95% CI: 0.803–0.926) in the training cohort and 0.856 (95% CI: 0.774–0.938) in the validation cohort, indicating good discriminative ability in both cohorts (Figure 2).

### 3.7 Calibration of the nomogram prediction model

**Figure 3:**
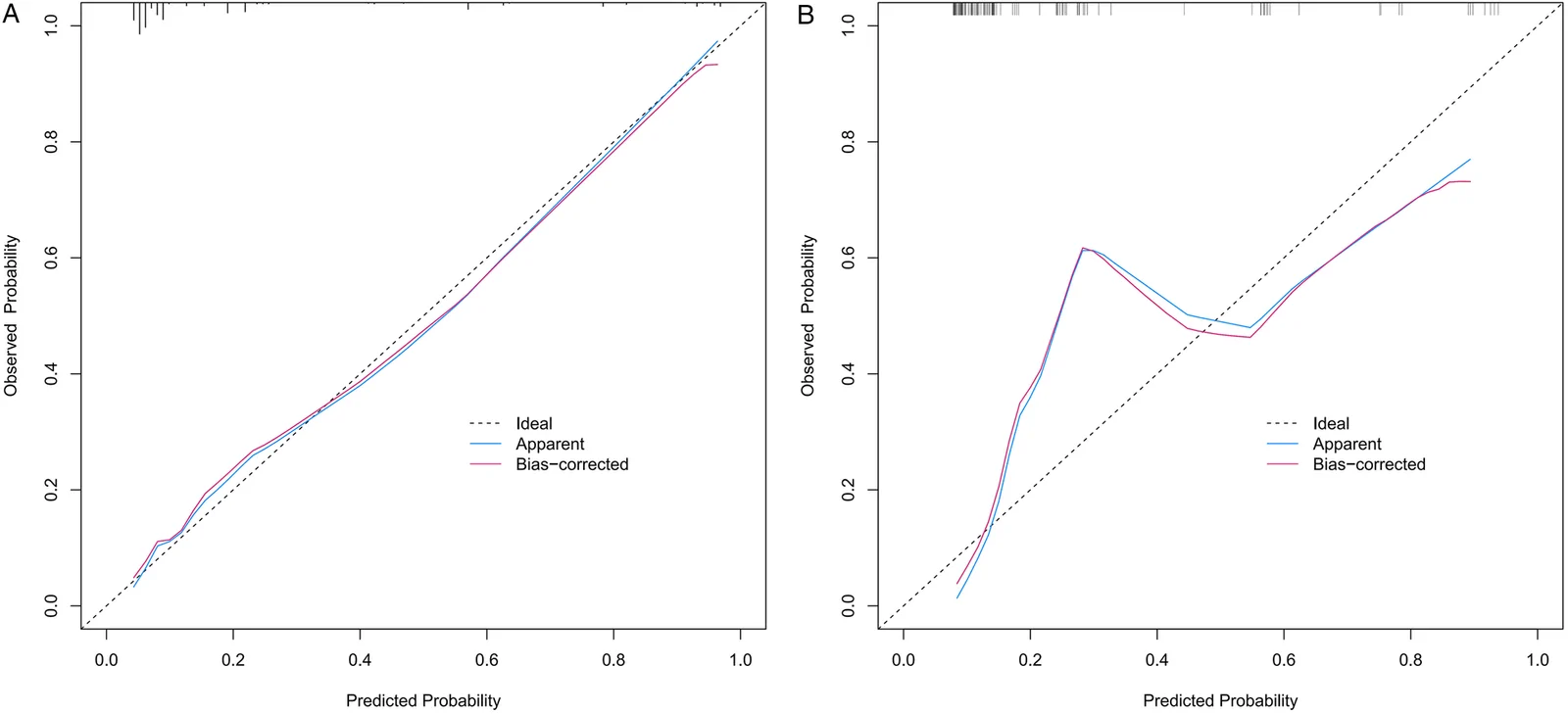
Calibration curves of the nomogram for predicting postoperative urinary dysfunction in the training cohort **(A)** and validation cohort **(B)** the dashed line represents the ideal prediction, the blue line represents the apparent prediction, and the pink line represents the bias-corrected prediction.

The calibration of the nomogram model was evaluated using calibration curves and the Hosmer-Lemeshow goodness-of-fit test. The calibration curves showed that the predicted probabilities were generally consistent with the observed probabilities in both the training and validation cohorts, and the apparent and bias-corrected curves were closely aligned, indicating good calibration of the model (Figure 3). The bias-corrected C-index was 0.863 in the training cohort and 0.857 in the validation cohort. The Hosmer-Lemeshow test showed *P* = 0.914 in the training cohort and *P* = 0.052 in the validation cohort, both of which were >0.05, indicating no statistically significant evidence of lack of fit in either cohort.

### 3.8 Clinical utility of the nomogram prediction model

**Figure 4:**
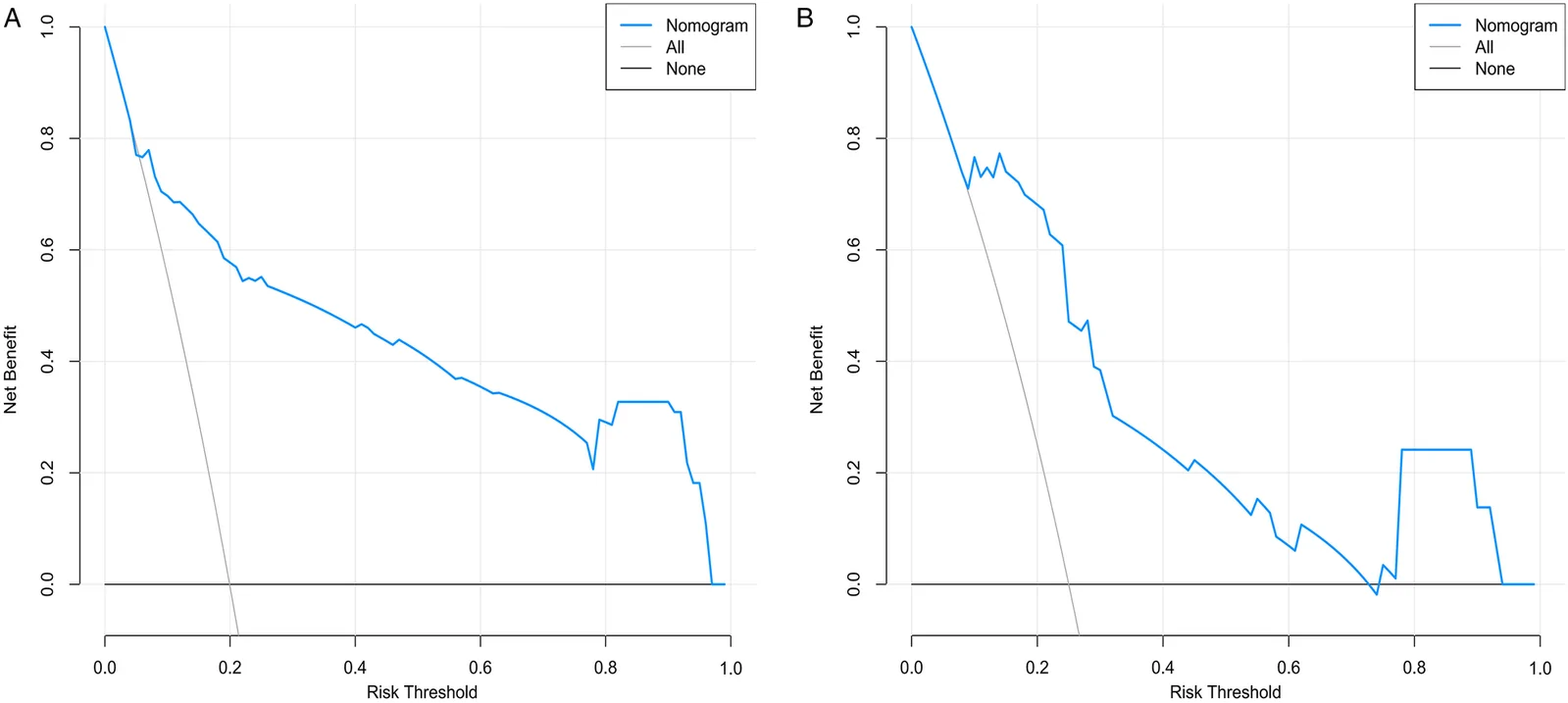
Decision curve analysis of the nomogram for predicting postoperative urinary dysfunction in the training cohort **(A)** and validation cohort **(B)** the blue line represents the nomogram model, the gray line represents the strategy of treating all patients, and the black line represents the strategy of treating no patients.

DCA was used to evaluate the clinical utility of the nomogram model. The results showed that the nomogram provided a higher net benefit than the “treat-all” or “treat-none” strategy across most threshold probabilities in both the training and validation cohorts, indicating favorable clinical applicability (Figure 4).

## 4 Discussion

With the development of the TME concept, laparoscopic techniques, and perioperative management strategies, oncological outcomes in patients with rectal cancer have continued to improve, and postoperative functional preservation and quality-of-life management have gradually become important components of comprehensive treatment (5, 21). Urinary dysfunction is an easily underestimated functional complication after rectal cancer surgery and may lead to urinary retention, failure of catheter removal, repeated catheterization, urinary tract infection, and prolonged hospital stay, thereby affecting patients’ postoperative recovery experience (10, 22). Previous studies have shown that urinary dysfunction after rectal cancer treatment is not uncommon (23). A prospective cohort study by Karlsson et al. found that symptoms such as urinary incontinence, bladder emptying difficulty, and urinary urgency increased after treatment compared with those at diagnosis (24). A recent systematic review also suggested that some patients still experience long-term urinary dysfunction, including urinary frequency, urinary retention, or urinary incontinence, more than 1 year after surgery (23). In the present study, the incidence of urinary dysfunction after laparoscopic radical surgery for rectal cancer was 21.4%, which is generally consistent with previous reports. Because the definition of urinary dysfunction, timing of assessment, surgical procedures included, and catheter removal strategies vary across studies, incidence rates should not be directly compared without caution.

Multivariable logistic regression analysis in this study showed that age ≥65 years, male sex, tumor distance from the anal verge ≤5 cm, intraoperative fluid rate ≥10 mL/kg/h, neoadjuvant chemoradiotherapy, documented incomplete pelvic autonomic nerve preservation, and postoperative urinary tract infection were independently associated with urinary dysfunction after laparoscopic radical surgery for rectal cancer. Age is an important baseline factor affecting postoperative recovery of urinary function. With increasing age, detrusor contractility may decline (25), and older patients may have relatively poor tolerance to anesthesia, analgesia, pelvic manipulation, and catheter removal; therefore, they are more prone to postoperative urinary retention, dysuria, or incomplete bladder emptying (18, 26). In the present study, patients aged ≥65 years had an increased risk of postoperative urinary dysfunction, which is generally consistent with previous findings. A systematic review and meta-analysis by Huang et al. on risk factors for postoperative urinary retention after colorectal surgery showed that older age was an important risk factor for postoperative urinary retention (27). In addition, the scoring system developed by Kim et al. for predicting bladder dysfunction after laparoscopic rectal cancer surgery also included age as a predictor (17). Therefore, in older patients with rectal cancer, postoperative catheter removal should not be based solely on a fixed time point; instead, individualized assessment should be performed by considering the patient's general condition, postoperative mobility, bladder filling sensation, and postvoid residual urine volume.

Male sex was also independently associated with postoperative urinary dysfunction in this study. One possible explanation is that the male pelvis is relatively narrow, which may make surgical exposure and deep pelvic manipulation more difficult during surgery for low rectal cancer, thereby increasing the risk of pelvic autonomic nerve traction or injury (12, 28). Previous studies on postoperative urinary retention after colorectal surgery have also identified male sex as a relevant risk factor. Grass et al. reported that male sex was an independent risk factor for urinary retention after colorectal surgery within an enhanced recovery pathway (29). Similarly, a meta-analysis by Huang et al. showed that male sex was associated with an increased risk of postoperative urinary retention after colorectal surgery (27). In the present study, patients with definite preoperative urinary dysfunction, long-term indwelling catheterization, neurogenic bladder, or severe benign prostatic hyperplasia requiring intervention were excluded; nevertheless, male sex remained associated with a higher risk. This finding suggests that sex-related differences may not be explained solely by overt urological diseases but may also be related to pelvic anatomical characteristics and surgical difficulty.

Tumor distance from the anal verge ≤5 cm was another independent factor associated with postoperative urinary dysfunction in the present study. Patients with tumors located ≤5 cm from the anal verge had a higher risk of postoperative urinary dysfunction than those with tumors located >5 cm from the anal verge. This finding has a clear anatomical and surgical basis. The operative field for low rectal cancer is closer to the pelvic floor and pelvic plexus, and surgical procedures often require sharp dissection, traction, and the use of energy devices in the narrow deep pelvis. Nerve preservation may be particularly challenging in patients with very low tumors, marked local inflammation, fibrosis after chemoradiotherapy, or severe pelvic adhesions (12, 13, 28, 30).

Neoadjuvant chemoradiotherapy was also independently associated with postoperative urinary dysfunction in the present study. Although neoadjuvant chemoradiotherapy plays an important role in local tumor control, treatment-related inflammation, edema, and pelvic fibrosis may obscure the anatomical planes and increase the technical difficulty of deep pelvic dissection and autonomic nerve preservation (30). A previous systematic review and meta-analysis involving 17,917 patients found that neoadjuvant therapy was associated with an increased risk of urinary incontinence compared with upfront surgery (OR = 1.31, 95% CI: 1.00–1.72; *P* = 0.05), although the potential influence of confounding factors, including surgical procedure and tumor stage, should be considered (31). Although urinary incontinence differs from the bladder-emptying dysfunction assessed in the present study, these findings suggest that neoadjuvant therapy may influence postoperative urinary outcomes. However, the present study did not separately evaluate specific neoadjuvant treatment regimens, radiation doses, treatment intervals, or the degree of pelvic fibrosis. Therefore, the observed association should be interpreted cautiously, and further studies are needed to clarify the effects of different neoadjuvant treatment strategies on postoperative urinary function.

Pelvic autonomic nerve preservation was one of the surgery-related factors most closely associated with postoperative urinary dysfunction in this study. During radical surgery for rectal cancer, the superior hypogastric nerves, inferior hypogastric nerves, pelvic splanchnic nerves, and pelvic plexus jointly participate in the regulation of bladder sensation, detrusor contraction, and urethral sphincter coordination (12, 15, 28). Although TME emphasizes complete mesorectal excision along the correct anatomical plane, low rectal cancer, local tumor invasion, a narrow operative field, and fibrosis after radiotherapy may increase the difficulty of autonomic nerve identification and preservation (12, 13, 28, 30). Kneist and Junginger reported that patients in whom the pelvic autonomic nerves were completely identified and preserved intraoperatively had significantly lower postoperative residual urine volume than those without complete preservation, suggesting that pelvic autonomic nerve preservation may help reduce the risk of postoperative bladder emptying dysfunction (32). In the present study, documented incomplete pelvic autonomic nerve preservation was associated with an increased risk of postoperative urinary dysfunction, further supporting the important role of standardized nerve preservation in functional outcomes after rectal cancer surgery. Because this was a retrospective study and nerve-preservation status was determined from operative records, some variability in intraoperative assessment and documentation may remain. Accordingly, this finding supports careful identification and preservation of the pelvic autonomic nerves during rectal cancer surgery but should not be interpreted as establishing a causal relationship.

This study found that an intraoperative fluid rate ≥10 mL/kg/h was associated with an increased risk of postoperative urinary dysfunction. Compared with total intraoperative fluid volume alone, fluid rate adjusted for body weight and operation time may better reflect the actual fluid load received by the patient. A previous meta-analysis on postoperative urinary retention after colorectal surgery showed that increased intraoperative fluid volume was one of the factors associated with postoperative urinary retention (27). In addition, ERAS guidelines and related reviews emphasize that perioperative fluid management in colorectal surgery should avoid fluid overload and advocate individualized or goal-directed fluid therapy (33, 34). Therefore, the findings of this study suggest that, during laparoscopic radical surgery for rectal cancer, unnecessary fluid load should be avoided while ensuring adequate circulatory perfusion, which may help reduce the risk of postoperative bladder overdistension, urinary retention, and delayed recovery of urinary function.

Postoperative urinary tract infection was also significantly associated with postoperative urinary dysfunction in this study. Urinary tract infection may affect voiding function after catheter removal through bladder mucosal inflammation, urethral irritation, urinary frequency, urgency, and painful urination. Conversely, dysuria, increased postvoid residual urine volume, repeated catheterization, or prolonged catheterization may also increase the risk of urinary tract infection; therefore, these two conditions may influence each other (35, 36). Kang et al., in a study including 93,931 patients undergoing surgery for colorectal cancer, showed that postoperative urinary tract infection and urinary retention are both urological complications that deserve attention after colorectal cancer surgery (10). The meta-analysis by Huang et al. also suggested that risk factors for postoperative urinary retention after colorectal surgery should be identified early and corresponding interventions should be implemented (27). In the present study, postoperative urinary tract infection had a high OR value but a wide confidence interval, suggesting that the effect estimate may have been influenced by the relatively small number of infection events. In addition, because urinary tract infection and urinary dysfunction may overlap in their temporal sequence, this variable may be more appropriate as a perioperative dynamic risk assessment indicator rather than a purely preoperative predictor.

Based on the results of multivariable logistic regression analysis, this study established a nomogram model incorporating seven factors: age, sex, tumor distance from the anal verge, intraoperative fluid rate, neoadjuvant chemoradiotherapy, pelvic autonomic nerve preservation, and postoperative urinary tract infection. The model showed good discriminative ability in both the training and validation cohorts, with AUC values of 0.864 and 0.856, respectively. Calibration was generally acceptable, although some deviation was observed in the validation cohort. The Hosmer–Lemeshow test showed no significant lack of fit in either cohort (*P* = 0.914 and *P* = 0.052, respectively), and DCA demonstrated clinical net benefit across a range of threshold probabilities. Because postoperative urinary tract infection was included in the model, the nomogram is intended primarily for perioperative dynamic risk assessment rather than exclusively preoperative prediction. Clinically, the model may assist in identifying patients at high risk for postoperative urinary dysfunction and support ongoing urinary catheter management, postvoid residual urine monitoring, bladder function training, and timely urological intervention.

This study has several limitations. First, this was a single-center retrospective study, and selection bias and information bias may have been present. Patients without sufficient operative documentation to determine pelvic autonomic nerve-preservation status were excluded. Nevertheless, the classification of nerve-preservation status was based on retrospective review of operative records and may have been influenced by differences in intraoperative assessment and documentation among surgeons. Second, the definition of urinary dysfunction in this study was mainly based on clinical intervention, postvoid residual urine volume, and catheterization status. Standardized assessments such as the International Prostate Symptom Score, bladder function questionnaires, or urodynamic examinations were not systematically included; therefore, mild or subjective urinary symptoms may have been underrecognized. Third, although internal validation was performed using training and validation cohorts, the validation cohort was derived from the same center, and external validation was lacking. Therefore, the generalizability of the model needs to be further evaluated in multicenter studies and different populations. Fourth, postoperative urinary tract infection was included as one of the final model variables. Although this may improve perioperative dynamic prediction, its temporal relationship with urinary dysfunction may overlap; thus, the model may be more suitable for early postoperative dynamic risk assessment. Future studies may further develop early prediction models based only on preoperative and intraoperative variables to better guide catheter management at an earlier stage. Fifth, this study did not further analyze potential factors such as neoadjuvant treatment regimens, anesthesia methods, analgesic medications, postoperative mobilization time, previous mild lower urinary tract symptoms, or diabetes duration. Future studies may incorporate these variables to further optimize model performance.

## 5 Conclusion

Age ≥65 years, male sex, tumor distance from the anal verge ≤5 cm, intraoperative fluid rate ≥10 mL/kg/h, neoadjuvant chemoradiotherapy, documented incomplete pelvic autonomic nerve preservation, and postoperative urinary tract infection were independently associated with urinary dysfunction after laparoscopic radical surgery for rectal cancer. The nomogram model constructed based on these factors demonstrated good discrimination, calibration, and clinical net benefit in both the training and validation cohorts, and may provide a reference for perioperative identification of high-risk patients, individualized catheter management, and postoperative follow-up.

## Data Availability

The raw data supporting the conclusions of this article will be made available by the authors, without undue reservation.
