# Supplementary material for: Risk factors and development of a nomogram prediction model for urinary dysfunction after laparoscopic radical surgery for rectal cancer
Source: Front Surg. 2026 Aug 11;13:1911971. doi: 10.3389/fsurg.2026.1911971 (PMC13539602; doi:10.3389/fsurg.2026.1911971)
Supplement: Supplementary file 1 [file Table1.docx]

**Supplementary Table 1.** Multicollinearity assessment of candidate predictors

| **Variable** | **VIF** | **1/VIF** |
| --- | --- | --- |
| Neoadjuvant chemoradiotherapy | 1.30 | 0.768 |
| Operation time | 1.28 | 0.780 |
| Tumor distance from the anal verge | 1.24 | 0.804 |
| Intraoperative blood loss | 1.20 | 0.830 |
| Pelvic autonomic nerve preservation | 1.19 | 0.841 |
| Intraoperative fluid rate | 1.15 | 0.869 |
| Postoperative urinary tract infection | 1.15 | 0.869 |
| Catheterization duration | 1.14 | 0.8759 |
| NLR | 1.12 | 0.892 |
| Age | 1.09 | 0.915 |
| Preoperative albumin | 1.08 | 0.922 |
| Diabetes mellitus | 1.07 | 0.936 |
| Sex | 1.03 | 0.969 |
| Mean VIF | 1.16 |  |

**Note:** VIF, variance inflation factor; NLR, neutrophil-to-lymphocyte ratio.
