# Supplementary material for: Risk factors and development of a nomogram prediction model for urinary dysfunction after laparoscopic radical surgery for rectal cancer
Source: Front Surg. 2026 Aug 11;13:1911971. doi: 10.3389/fsurg.2026.1911971 (PMC13539602; doi:10.3389/fsurg.2026.1911971)
Supplement: Supplementary file 2 [file Table2.docx]

**Supplementary Table 2.** Point assignment of the nomogram

| **Variable** | **Category** | **Points** |
| --- | --- | --- |
| **Age** | <65 years | 0 |
|  | ≥65 years | 57.5 |
| **Sex** | Female | 0 |
|  | Male | 34.9 |
| **Tumor distance from the anal verge** | ≤ 5cm | 35.9 |
|  | ＞5cm | 0 |
| **Intraoperative fluid rate** | <10 mL/kg/h | 0 |
|  | ≥10 mL/kg/h | 63.5 |
| **Neoadjuvant chemoradiotherapy** | No | 0 |
|  | Yes | 40.6 |
| **Pelvic autonomic nerve preservation** | Documented preservation | 0 |
|  | Incomplete preservation | 62.6 |
| **Postoperative urinary tract infection** | No | 0 |
|  | Yes | 100 |
